# The controversial nature of the *Weissella* genus: technological and functional aspects versus whole genome analysis-based pathogenic potential for their application in food and health

**DOI:** 10.3389/fmicb.2015.01197

**Published:** 2015-10-27

**Authors:** Hikmate Abriouel, Leyre Lavilla Lerma, María del Carmen Casado Muñoz, Beatriz Pérez Montoro, Jan Kabisch, Rohtraud Pichner, Gyu-Sung Cho, Horst Neve, Vincenzina Fusco, Charles M. A. P. Franz, Antonio Gálvez, Nabil Benomar

**Affiliations:** ^1^Área de Microbiología, Departamento de Ciencias de la Salud, Facultad de Ciencias Experimentales, Universidad de Jaén, Jaén, Spain; ^2^Department of Microbiology and Biotechnology, Federal Research Institute of Nutrition and Food, Max Rubner-Institut, Kiel, Germany; ^3^Institute of Sciences of Food Production, National Research Council of Italy, Bari, Italy

**Keywords:** *Weissella*, *in silico* analysis, genome, virulence, antibiotic resistance

## Abstract

Despite the use of several *Weissella (W.)* strains for biotechnological and probiotic purposes, certain species of this genus were found to act as opportunistic pathogens, while strains of *W. ceti* were recognized to be pathogenic for farmed rainbow trout. Herein, we investigated the pathogenic potential of weissellas based on *in silico* analyses of the 13 whole genome sequences available to date in the NCBI database. Our screening allowed us to find several virulence determinants such as collagen adhesins, aggregation substances, mucus-binding proteins, and hemolysins in some species. Moreover, we detected several antibiotic resistance-encoding genes, whose presence could increase the potential pathogenicity of some strains, but should not be regarded as an excluding trait for beneficial weissellas, as long as these genes are not present on mobile genetic elements. Thus, selection of weissellas intended to be used as starters or for biotechnological or probiotic purposes should be investigated regarding their safety aspects on a strain to strain basis, preferably also by genome sequencing, since nucleotide sequence heterogeneity in virulence and antibiotic resistance genes makes PCR-based screening unreliable for safety assessments. In this sense, the application of *W. confusa* and *W. cibaria* strains as starter cultures or as probiotics should be approached with caution, by carefully selecting strains that lack pathogenic potential.

## Introduction

*Weissella* species are non-spore forming, catalase-negative and Gram-positive bacteria that are non-motile, with the exception of *Weissella (W.) beninensis*. To date, the *Weissella* genus comprises 19 validly described species (February 2015^1^). Most of these were isolated from and associated with fermented foods, e.g., *W. confusa*, *W. cibaria* (Björkroth et al., 2002), *W. kimchii* (Choi et al., 2002), *W. koreensis* (Lee et al., 2002), and *W. viridescens* (Comi and Iacumin, 2012). *Weissella viridescens* is considered the type species of the *Weissella* genus (Collins et al., 1994). As a member of the lactic acid bacteria (LAB), *Weissella* have complex nutritional requirements. Because of this, they inhabit nutrient-rich environments and can be isolated from a variety of such sources, including vegetables, meat, fish, raw milk, sewage, blood, soil, the gastrointestinal tracts of humans and animals, as well as the oral cavity and uro-genital tract of humans (Fusco et al., 2015).

From a technological point of view, *Weissella* plays an important role in fermentation processes such as the production of silage, as well as in food fermentations based on vegetables or meat as substrate (Björkroth et al., 2002; Santos et al., 2005).

Several weissellas, mainly belonging to the *W. confusa* and *W. cibaria* species, are being extensively studied for their ability to produce significant amounts of non-digestible oligosaccharides and extracellular polysaccharides, which can be used as prebiotics or for other applications in food, feed, clinical, and cosmetics industries.

Furthermore, several *Weissella* strains have been found to act as probiotics, mainly due to their antimicrobial activity, as is the case for certain bacteriocinogenic strains of *W. paramesenteroides*, *W. hellenica*, and *W. cibaria* (Fusco et al., 2015). For example, *W. hellenica* DS-12 isolated from flounder intestine has been used as probiotic in fish, due to its antimicrobial activity against fish pathogens, such as *Edwardsiella*, *Pasteurella*, *Aeromonas*, and *Vibrio* (Cai et al., 1998). Also, strains of *W. cibaria* were proposed as probiotics for oral health, inhibiting *Streptococcus mutans* glucan biofilm formation (Kang et al., 2006). Recently, weissellas were also shown to exhibit chemopreventive and anti-tumor effects (Kwak et al., 2014).

On the detrimental side, some weissellas were reported to be involved in disease outbreaks such as otitis, sepsis, endocarditis, and even fish mortality (Flaherty et al., 2003; Harlan et al., 2011; Lee et al., 2011; Welch and Good, 2013). Human infections caused by *Weissella* spp. are, however, rarely reported, and occur mostly in patients with impaired host defenses (Lee et al., 2011). Curiously, therefore, in the same species both beneficial and detrimental strains can be found. As an example, *W. confusa* causes sepsis and other serious infections in humans and animals, while it has a functional role in food fermentations and has also been suggested as a probiotic (Fusco et al., 2015).

The probiotic and pro-technological potential of weissellas therefore collides with the potential of these bacteria as human pathogens. As for enterococci, whose use for food and health application has been controversial (Franz et al., 2003; Ogier and Serror, 2008), a safety assessment of each strain that is intended to be used as starter culture or as probiotic, should thus be recommended.

Whole genome sequencing and sequence annotation is increasingly being used as valuable tool for assessing microbial food quality and safety aspects (Alkema et al., 2015), allowing the identification of new genes that may have an important impact on cell metabolism, fitness, and virulence.

Herein, we report the investigation of the pathogenic potential of weissellas based on *in silico* analyses of the 13 whole genome sequences (of 13 strains belonging to nine *Weissella* species) to date available.

## Materials and Methods

### Data Sequences

**FIGURE 1:**
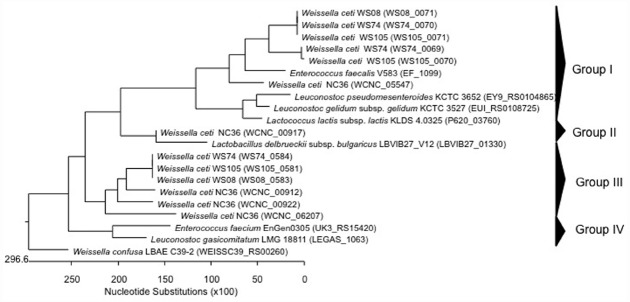
**Phylogenetic relationships of ***Weissella*** species and ***Leuconostoc Lactobacillus Enterococcus*** inferred from the alignment of collagen adhesin encoding genes.** The sequences were aligned and the most parsimonious phylogenetic trees were constructed using the CLUSTAL W of Lasergene program, version 5.05 (MegAlign, Inc., Madison, WI, USA). The scale below indicates the number of nucleotide substitutions. Accession numbers are indicated in parentheses.

**FIGURE 2:**
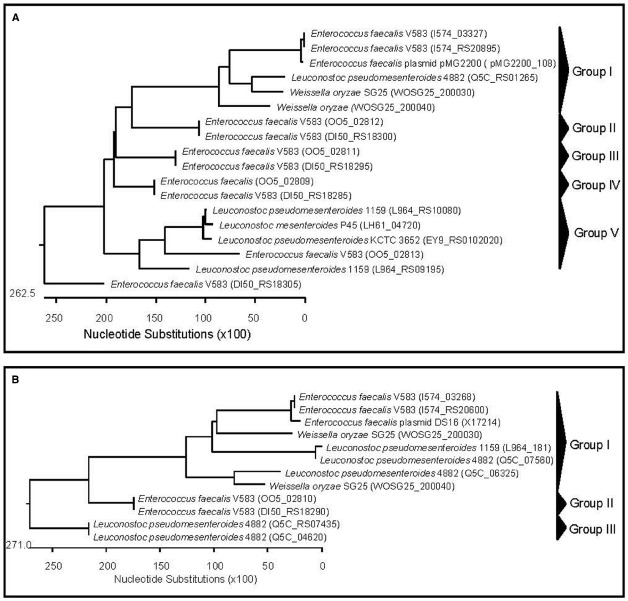
**Phylogenetic relationships of ***Weissella*** species and ***Leuconostoc Enterococcus*** inferred from the alignment of aggregation substances (A) and aggregation substance Asa1/PrgB (B) encoding genes.** The sequences were aligned and the most parsimonious phylogenetic trees were constructed using the CLUSTAL W of Lasergene program, version 5.05 (MegAlign, Inc., Madison, WI, USA). The scale below indicates the number of nucleotide substitutions. Accession numbers are indicated in parentheses.

**FIGURE 3:**
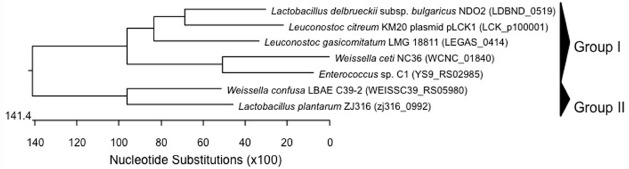
**Phylogenetic relationships of ***Weissella*** species and ***Leuconostoc*** Lactobacillus Enterococcus inferred from the alignment of mucus-binding protein encoding genes.** The sequences were aligned and the most parsimonious phylogenetic trees were constructed using the CLUSTAL W of Lasergene program, version 5.05 (MegAlign, Inc., Madison, WI, USA). The scale below indicates the number of nucleotide substitutions. Accession numbers are indicated in parentheses.

**FIGURE 4:**
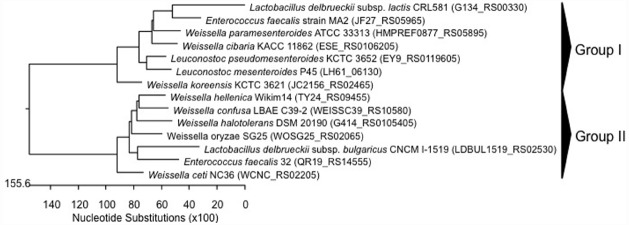
**Phylogenetic relationships of ***Weissella*** species and ***Leuconostoc*** Lactobacillus Enterococcus inferred from the alignment of multidrug transporter involved in fosfomycin resistance encoding genes.** The sequences were aligned and the most parsimonious phylogenetic trees were constructed using the CLUSTAL W of Lasergene program, version 5.05 (MegAlign, Inc., Madison, WI, USA). The scale below indicates the number of nucleotide substitutions. Accession numbers are indicated in parentheses.

**FIGURE 5:**
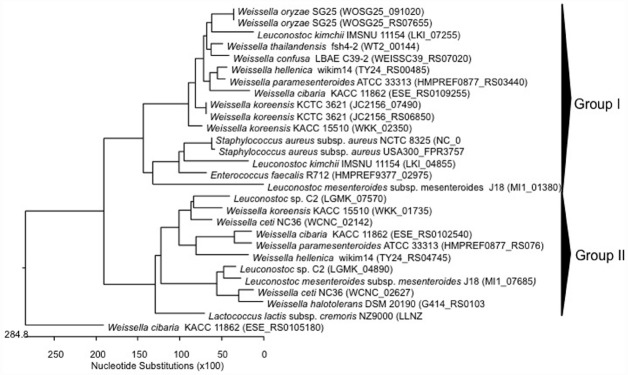
**Phylogenetic relationships of ***Weissella*** species and ***Leuconostoc*** Lactobacillus Enterococcus inferred from the alignment of methicillin resistance protein encoding genes.** The sequences were aligned and the most parsimonious phylogenetic trees were constructed using the CLUSTAL W of Lasergene program, version 5.05 (MegAlign, Inc., Madison, WI, USA). The scale below indicates the number of nucleotide substitutions. Accession numbers are indicated in parentheses.

Data sequences of genomes of the 13 strains belonging to nine *Weissella* species (Table 1) were retrieved from the National Centre for Biotechnology Information (NCBI^2^; accessed on February, 2015). All genome sequences available were analyzed for the presence of different virulence determinants (aggregation substances, adhesins, toxins, pili, hemolysins) and of antibiotic resistance genes, such as fosfomycin and methicillin resistance genes. The accession numbers of each target gene sequence are indicated in Tables 1–3 and Figures 1–5.

**TABLE 1:** **Genome characteristics of *Weissella* species isolated from different sources**.

**Strains**	**Genome size (bp)**	**Source**	**RefSeq/GenbankWGS**
*Weissella ceti* WS08	1.355.850	Fish brain	NZ_CP007588.1
*Weissella ceti* WS74	1.389.510	Fish brain	NZ_CP009223.1
*Weissella ceti* WS105	1.390.400	Fish brain	NZ_CP009224.1
*Weissella ceti* NC36	1.352.640	Fish spleen	NZ_ANCA00000000.1
*Weissella cibaria* KACC 11862	2.320.000	Kimchi	NZ_AEKT00000000.1
*Weissella confusa* LBAE C39-2	2.280.000	Wheat sourdough	NZ_CAGH00000000.1
*Weissella halotolerans* DSM 20190	1.360.000	Sausage	NZ_ATUU00000000.1
*Weissella hellenica* Wikim14	1.920.000	Kimchi	BBIK00000000.1
*Weissella koreensis* KACC 15510	1.441.470	Kimchi	NC_015759.1
*Weissella koreensis* KCTC 3621	1.728.940	Kimchi	NZ_AKGG00000000.1
*Weissella oryzae* SG25	2.130.000	Fermented rice grains	NZ_BAWR00000000.1
*Weissella paramesenteroides* ATCC 33313	1.962.173	Human	NZ_ACKU00000000.1
*Weissella thailandensis* fsh4-2	1.968.992	Jeotkal (Korean fermented fish condiment)	HE575133 to HE575182

### Phylogenetic Analyses

All selected target genes were subjected to phylogenetic analyses to determine phylogenetic relationships with those of closely related genera. Alignment of sequences was done using the CLUSTAL W module of the Lasergene program, version 5.05 (MegAlign, Inc., Madison, WI, USA). Phylogenetic trees were reconstructed by the maximum parsimony method using MegAlign (Lasergene program, version 5.05).

## Results and Discussion

### Virulence Determinants in *Weissella*

The safety of many LAB species have been recognized as GRAS (Generally Regarded As Safe; for the USA; Food and Drug Administration, 1999) or have attained the QPS (*Qualified Presumption of Safety*; for the European Commission; European Food Safety Authority (EFSA), 2004) status. Indeed, many people refer to these bacteria as being innocuous, and even associated with health beneficial properties. However, it is well known that certain species within a genus, or even certain strains within a specific species, may have different health impacts, as has been pointed out for the enterococci (Franz et al., 2003; Ogier and Serror, 2008). So far, no *Weissella* species have QPS status (European Food Safety Authority (EFSA), 2015) Data on virulence factors present in *Weissella* species are quite scarce, and genomic analysis can therefore aid in detecting and describing the occurrence of virulence determinants that may be present at species or strain level within this genus. In this regard, Ladner et al. (2013) found in the genome sequence of *W. ceti* NC36, an emerging pathogen of farmed rainbow trout in the United States, the presence of several putative virulence factor genes, which did not have homologs encoded in any of the other sequenced *Weissella* genomes. In particular, these include five collagen adhesin genes (WCNC_00912, WCNC_00917, WCNC_00922, WCNC_05547, and WCNC_06207), a platelet-associated adhesin gene (WCNC_01820) and a gene for a mucus-binding protein (WCNC_01840; Ladner et al., 2013). The five collagen adhesin genes included those from *W. ceti* WS105 (three collagen adhesins), *W. ceti* WS74 (three collagen adhesins), *W. ceti* WS08 (two collagen adhesins), and *W. confusa* LBAE C39-2 (one collagen adhesin; Table 2). DNA sequences coding for collagen adhesin proteins in *Weissella* spp. and other LAB (*Leuconostoc* “*Ln.*”, *Lactococcus* “*Lc.*”, *Enterococcus* “*E.*”, and *Lactobacillus* “*Lb.*”) were aligned using CLUSTAL W of the Lasergene program, version 5.05 (MegAlign, Inc., Madison, WI, USA) and the most parsimonious phylogenetic tree was reconstructed. The dendrogram generated showed four groups (Figure 1). The first group contained two of the three collagen adhesin genes found in *W. ceti* strains, *E. faecalis*, *Leuconostoc* and *Lactococcus*, while genes for group II collagen adhesins were only present in *W. ceti* NC36 and *Lb. delbrueckii*. The third group presented the genes for the third collagen produced by *W. ceti* strains. In the last group (group IV) *E. faecium* and *Leuconostoc* collagen adhesin genes clustered together, however a *W. confusa* LBAE C39-2 collagen adhesin gene also formed a single lineage (Figure 1). In general, the close relatedness of *Weissella*, *Enterococcus*, and *Leuconostoc* collagen adhesion genes at the level of the primary protein sequence may suggest the same evolutionary origin of these proteins in these bacteria. Further genome sequencing of other *Weissella* species would be required to analyze this relatedness with enterococci and leuconostocs in more depth. The presence of virulence determinants commonly present in enterococci also in weissellas and leuconostocs may suggest a horizontal gene transfer event from the *Enterococcus* genus, the latter of which indeed includes strains that are commonly present in many different habitats (Franz et al., 2003).

**TABLE 2:** **Potential virulence genes of *Weissella* species as revealed by *in silico* screening of the annotated genome sequences**.

**Strains**		**Virulence factors detected by analysis *in silico* of genome sequences (locus_tag)**			
	**Aggregation substance**	**Collagen adhesion**	**Hemolysin**	**Mucus-binding proteins**	**Staphylococcal surface protein**
*W. ceti* WS08	–	2 CA (WS08_0071, WS08_0583)	1 Hly (WS08_0556), 1 HlyA (WS08_0902)	–	5SPA (WS08_0360, WS08_0450, WS08_0978, WS08_1156, WS08_1190)
*W. ceti* WS74	–	3 CA (WS74 0069, WS74 0070, WS74_0584)	1 Hly (WS74_0557), 1 HlyA (WS74_0968)	–	4 SPA (WS74_0360, WS74_0451, WS74_1225, WS74_1261)
*W. ceti* WS105	–	3 CA (WS105 0070, WS105 0071, WS105_0581)	1 Hly (WS105_0554), 1 HlyA (WS105_0965), 1 Hly-like protein (WS105_0227)	–	4 SPA (WS105_0358, WS105_0448, WS105_1219, WS105_1255)
*W. ceti* NC36	–	5 CA (WCNC 00912, WCNC 00917, WCNC 00922, WCNC 05547, WCNC 06207) 1 PA-ADHE	–	1 MBP (WCNC_01840)	–
*W. cibaria* KACC 11862	–	–	2 Hly (ESE_RS0108795, ESE_RS11605)	–	–
*W. confusa* LBAE C39-2	–	1 (WEISSC39_RS00260)	2 Hly (WEISSC39_RS08935, WEISSC39_RS08940)	1 MBP (WEISSC39_RS05980)	–
*W. halotolerans* DSM 20190	–	–	2 Hly (G414_RS0106810, G414_RS0106815)	–	–
*W. hellenica* Wikim14	–	–	2 Hly (TY24_RS08990, TY24_RS08995)	–	–
*W. koreensis* KACC 15510	–	–	–	–	–
*W. koreensis* KCTC 3621	–	–	2 Hly (JC2156_RS08435, JC2156_10680)	–	–
*W. oryzae* SG25	2 AGS (WOSG25_200030, WOSG25_200040) 2 Asa1/PrgB (WOSG25_200030, WOSG25_200040)	–	3 hemolysins (WOSG25 150280, WOSG25_RS09625, WOSG25_RS09635)	–	–
*W. paramesenteroides* ATCC 33313	–	–	2 Hly (HMPREF0877_RS09665, HMPREF0877_RS09670)	–	–
*W. thailandensis* fsh4-2	–	–	2 Hly (WT2_01519, WT2_01520)		–

The role of the genes above in the virulence of *Weissella* is still unknown. Further studies are needed to confirm whether these virulence genes are expressed or not, and if they are located on mobile genetic elements (e.g., transposons). However, the collagen adhesin protein (Ace) in *Enterococcus* was reported to be involved in adhesion to collagen and to contribute to infective endocarditis and urinary tract infection (Aymerich et al., 2006; Nallapareddy et al., 2011). In addition, the common localization of this gene on plasmids or transposon may pose a risk related with horizontal gene transfer to pathogenic bacteria. On the other hand, adherence is an important pre-requisite for the colonization of probiotics, providing a competitive advantage in different ecosystems. The presence of adhesin genes in weissellas that are not located on mobile genetic elements, and which are not transferable, should therefore not negatively reflect on the strains probiotic potential, and should not be an excluding factor for its use. It could be regarded as a key factor for the attachment of probiotic bacterial cell in the human host, and thus could be regarded as a colonization factor rather than a virulence factor (Dunne et al., 2004).

*In silico* analyses of *Weissella* genomes also showed the presence of genes coding for two unnamed aggregation substances (WOSG25_050600 and WOSG25_190240) as well as two aggregation substances named Asa1/PrgB (WOSG25_200030 and WOSG25_200040) in *W. oryzae* SG25 (Table 2). The sequences of the genes encoding the unnamed aggregation substances, the aggregation substances Asa1/PrgB from *W. oryzae* SG25 and those of other LAB (*Leuconostoc* and *Enterococcus*) were aligned using the CLUSTAL W of Lasergene program, and the most parsimonious phylogenetic tree was constructed. The results obtained showed that the aggregation substance genes found in *W. oryzae* SG25 clustered with those of *Ln. pseudomesenteroides* and *E. faecalis* (group I in dendrogram), the latter being the aggregation substance gene of *E. faecalis* pMG2200, which is located on a plasmid. This suggests that horizontal gene transfer from *E. faecalis* to other LAB such as *Weissella* and *Leuconostoc* (Figure 2A) probably occurred. Other aggregation substance genes of different strains of *E. faecalis*, *Ln. mesenteroides*, and *Ln. pseudomesenteroides* were very divergent, forming several clusters (Figure 2A). When aggregation substance gene sequences of Asa1/PrgB from *W. oryzae* SG25, *E. faecalis*, and *Ln. pseudomesenteroides* were clustered, the dendrogram showed aggregation substances Asa1/PrgB coding genes from *W. oryzae* SG25 and *E. faecalis* or *Leuconostoc* (Group I) to cluster very closely, also suggesting a horizontal gene transfer between these bacteria (Figure 2B). The presence of an *asa1* gene was also reported in other potential probiotic LAB, such as *Lb. casei* SJRP35, *Ln. citreum* SJRP44, and *Ln. mesenteroides* subsp. *mesenteroides* SJRP58 isolated from Water-Buffalo Mozzarella Cheese (Jeronymo-Ceneviva et al., 2014), and also in *Lc. lactis* subsp. *Lactis* KT2W2L (Hwanhlem et al., 2013). As for adhesins, aggregation substances are considered virulence factors. However, the safety assessment of strains harboring adhesins or aggregation substances may highly depend on the localization of such genes on mobile genetic elements and on other safety aspects of the strain. However, the difference in infectivity between weissellas that are either probiotic or involved in infections such as endocarditis, cannot be explained by differences in adherence potential alone, but other factors involved in promoting disease must exist in pathogenic strains, as was similarly postulated by Vankerckhoven et al. (2007) for lactobacilli involved in human infections.

Genes for mucus-binding protein (Mub), which can serve as effector molecules involved in mechanisms of adherence of bacteria to the host, were detected in *W. ceti* NC36 and *W. confusa* LBAE C39-2 (Table 2) genome sequences. The phylogenetic tree of nucleotide sequences encoding mucus-binding proteins of weissellas, as well as of the closely related genera *Leuconostoc*, *Lactobacillus*, and *Enterococcus*, showed that the *W. ceti* NC36 *mub* gene clustered closely with *Enterococcus* sp. C1 *mub* in the same group together with sequences from *Lb. delbrueckii* and *Leuconostoc*. However, the *mub* of *W. confusa* LBAE C39-2 clustered with that of *Lb. plantarum* (Figure 3). The presence of mucus-binding protein may be a desirable feature in probiotic bacteria, as it may play an important role in the adhesion of the probiotic strain to host surfaces (Mack et al., 1999). However, this property is obviously problematic in potentially pathogenic strains.

Regarding other virulence factors, such as the Serine-rich adhesin for platelets, which is also important in bacterial adhesion and is considered the central event in the pathogenesis of infective endocarditis (Sullam, 1994), this adhesin was only detected in the pathogen *W. ceti* NC36 (WCNC_RS02030; Table 2). On the other hand, the gene encoding the Serine-rich adhesin for platelets was detected also in probiotic lactobacilli such as *Lb. johnsonii* NCC533 and *Lb. reuteri* (Zhou and Wu, 2009), but not in leuconostocs. Concerning the staphylococcal surface protein A, which is involved in invasion and infectivity, several genes coding for a similar protein were detected in *W. ceti* WS08 (WS08_0360, WS08_0450, WS08_0978, WS08_1156, WS08_1190), *W. ceti* WS74 (WS74_0360, WS74_0451, WS74_1225, WS74_1261), and *W. ceti* WS105 (WS105_0358, WS105_0448, WS105_1219, WS105_1255). However, no homologous genes were detected in either leuconostocs or in lactobacilli (Table 2). Due to the low homology of virulence determinants in weissellas, it is difficult to detect such genes by PCR, thus genome sequencing would be the only way to detect and identify these determinants.

The presence of genes for hemolysin or hemolysin-like proteins in weissellas was revealed by *in silico* analysis of the annotated genomes sequences (Table 2). The ubiquitous occurrence of such genes in probiotic lactobacilli and bifidobacteria, and also in other LAB such as leuconostocs, may raise questions about their function and virulence potential. The role of these genes in LAB virulence is unknown, and their presence in the genome of a strain intended to be used as probiotic should not necessarily be an exclusion factor for targeting strains for probiotic use. As shown in Table 2, weissellas harbored in their genome 1–3 hemolysin encoding genes, i.e., genes for hemolysin A and also hemolysin-like proteins.

### Antibiotic Resistance

Studies on antibiotic resistance profiles in *Weissella* genus are very limited, and MIC breakpoints have not been defined by EFSA. Thus, to categorize weissellas as sensitive or resistant to different antibiotics of clinical relevance is a difficult task. These bacteria are known for their intrinsic resistance to antibiotics inhibiting cell wall biosynthesis such as vancomycin and fosfomycin, similar to other Gram-positive bacteria (Arca et al., 1997), and also to antibiotics that inhibit tetrahydrofolate biosynthesis such as sulfamethoxazol and trimethoprim (Liu et al., 2009). Furthermore, resistance to gentamicin, kanamycin, and norfloxacin was also reported in food-associated weissellas (Patel et al., 2012).

Reports about molecular detection of antibiotic resistance genes in weissellas are very scarce, possibly due to the high divergence of resistance genes. In this sense, only *mef* (A/E) drug efflux pump genes involved in the active efflux of macrolides (Clancy et al., 1996; Tait-Kamradt et al., 1997) were detected in *W. cibaria* of aquatic origin (Muñoz-Atienza et al., 2013). When we analyzed the *in silico* genome sequences of *Weissella* spp., different antibiotic resistance genes were detected, such as those coding for fosfomycin and methicillin resistance proteins in almost all the genomes of *Weissella* strains sequenced (Table 3) to date. For example, the *fosB* gene coding for fosfomycin resistance was detected in *W. ceti* WS08 (WS08_1256), *W. ceti* WS74 (WS74_1327) and *W. ceti* WS105 (WS105_1321), and nucleotide sequences of the genes were identical in all cases. In addition, other weissellas harbored multidrug transporters involved in fosfomycin and deoxycholate resistance, as was the case for *W. ceti* NC36 (WCNC_RS02205), *W. cibaria* KACC 11862 (ESE_RS0106205), *W. confusa* LBAE C39-2 (WEISSC39_RS10580), *W. halotolerans* DSM 20190 (G414_RS0105405), *W. hellenica* Wikim14 (TY24_RS09455), *W. koreensis* KCTC 3621 (JC2156_RS02465), *W. oryzae* SG25 (WOSG25_RS02065), and *W. paramesenteroides* ATCC 33313 (HMPREF0877_RS05895; Table 3). An alignment of nucleotide sequences encoding multidrug transporters involved in fosfomycin resistance of weissellas and other LAB by CLUSTAL W alignment showed sequences to cluster into two groups. High similarities in sequences again suggested an evolutionary relationship of weissellas, leuconostocs, enterococci, and lactobacilli (Figure 4) sequences. Fosfomycin is a broad antibacterial agent that targets several pathogens such as *Haemophilus* spp., *Staphylococcus* spp. and most of the enteric gram-negative bacteria. Although no cross resistances are known to occur for other antibiotics, there are no data on whether or not the *fosB* gene is transferable to other bacteria. Thus, weissellas harboring the *fosB* gene should be investigated in terms of the transferability of this gene, especially to pathogens. In the case of enterococci, these bacteria contain a conjugative plasmid that harbors the novel *fosB* transposon (IS*L3*-like transposon), as well as the Tn*1546*-like transposon (containing *vanA* and *fosB*, Qu et al. (2014)). Should weissellas harbor no transferable *fosB* gene, a potential probiotic use of these bacteria should be possible, especially when infection treatment with a combination of other antibiotics is possible.

**TABLE 3:** **Antibiotic resistance genes of *Weissella* species as revealed by *in silico* screening of the annotated genome sequences**.

**Strains**	**Antibiotic resistance determinants detected by analysis *in silico* of genome sequences (locus_tag)**					
	**Daunorubicin**	**Fosfomycin**	**Methicillin**	**Glycopeptide**	**Sulfonamide**	**Tetracycline**
*W. ceti* WS08	–	1 FosB (WS08_1256)	–	–	Sul (WS08_0966)	–
*W. ceti* WS74	–	1 FosB (WS74_1327)	–	–	Sul (WS74_1032)	–
*W. ceti* WS105	–	1 FosB (WS105_1321)	–	–	Sul (WS105_1028)	Tet (WS105_0392)
*W. ceti* NC36	–	1 MDT-FosB (WCNC_RS02205)	2 MRP (WCNC_02142, WCNC_02627)	–	–	–
*W. cibaria* KACC 11862	–	1 MDT-FosB (ESE_RS0106205)	3 MRP (ESE_RS0109255, ESE_RS0102540, ESE_RS0105180)	VanZ (ESE_RS0111030)	–	–
*W. confusa* LBAE C39-2	–	1 MDT-FosB (WEISSC39_RS10580)	1 MRP (WEISSC39_RS07020)	VanZ (WEISSC39_RS04975)	–	–
*W. halotolerans* DSM 20190	1 DrrC (G414_RS0101040)	1 MDT-FosB (G414_RS0105405)	1 MRP (G414_RS0103120)	–	–	–
*W. hellenica* Wikim14	1 DrrC (TY24_RS06500)	1 MDT-FosB (TY24_RS09455)	2 MRP (TY24_RS04745, TY24_RS00485)	–	–	–
*W. koreensis* KACC 15510	–	–	2 MRP (WKK_01735, WKK_02350)	–	–	–
*W. koreensis* KCTC 3621	–	1 MDT-FosB (JC2156_RS02465)	2 MRP (JC2156_RS06850, JC2156_07490)	–	–	–
*W. oryzae* SG25	1 DrrC (WOSG25_RS07165)	1 MDT-FosB (WOSG25_RS02065)	2 MRP (WOSG25_091020, WOSG25_RS07655)	–	–	–
*W. paramesenteroides* ATCC 33313	–	1 MDT-FosB (HMPREF0877_RS05895)	2 MRP (HMPREF0877_RS07670, HMPREF0877_RS03440)	VanZ (HMPREF0877_1234)	–	–
*W. thailandensis* fsh4-2	–	–	1 MRP (WT2_00144)	–	–	Tet (WT2_00189)
*Weissella* sp.	ND	ND	ND	ND	ND	*sul1, sul2* genes*

DrrC, daunorubicin resistance protein; FosB, fosfomycin resistance protein; MDT-FosB, multidrug transporter involved in fosfomycin resistance; MRP, methicillin resistance protein; Sul, sulfonamide resistance protein; Tet, tetracycline resistance protein; VanZ, glycopeptide resistance protein. *Byrne-Bailey et al. (2009).

The intrinsic resistance of weissellas toward vancomycin may be attributed to the absence of D-Ala-D-lactate in their cell wall, which is the target of vancomycin. A similar situation exists in other LAB such as *Lactobacillus*, *Pediococcus*, and *Leuconostoc* species (Ammor et al., 2007). Thus, such resistance cannot be attributed to acquisition of resistance genes. Nevertheless, some weissellas harbored in their genomes the *vanZ* resistance gene, as is the case for *W. cibaria* KACC 11862 (ESE_RS0111030), *W. confusa* LBAE C39-2 (WEISSC39_RS04975), and *W. paramesenteroides* ATCC 33313 (HMPREF0877_1234). This gene confers resistance to teicoplanin and does not involve the incorporation of a substituent of D-alanine into the peptidoglycan precursors (Arthur et al., 1995; Table 3).

No data have been reported on methicillin resistance to date. However, analyzing the *in silico* genome sequences of weissellas available in NCBI database, methicillin resistance protein encoding genes were detected in all weissellas (sequenced genomes of nine species; Table 3). Methicillin resistance proteins are found in *Staphylococcus* spp. and especially in *S. aureus*, and the presence of genes encoding such proteins in *Weissella* species may suggest a horizontal gene transfer between the genera. The alignment of nucleotide sequences for methicillin resistance protein in weissellas and other Gram-positive bacteria (*S. aureus*, *Leuconostoc*, *E. faecalis*, and *Lactococcus*) clearly showed an evolutionary relationship between the methicillin-resistance sequences of *Weissella* and the other Gram-positive bacteria *S. aureus*, *Leuconostoc*, and *E. faecalis* (Figure 5). The dendrogram showed two main groups, in which the genes from *Weissella* spp. were distributed regardless of the species, strain or origin. A divergence of methicillin resistance genes could even be observed in a single strain of *W. ceti* NC36, which carried multiple methicillin resistance genes, varying considerably in nucleotide sequence (Figure 5). On the other hand, one of two genes encoding for methicillin resistance in *W. cibaria* KACC 11862 (ESE_RS0105180) formed a single lineage, as such being divergent from the other weissellas and other Gram-positive bacteria (Figure 5). Further studies should elucidate the functionality of the methicillin resistance genes of weissellas, and the transferability of these genes to other bacteria.

Sulfonamide resistance could be due either to mutation in the chromosomal gene that mediates dihydropteroate synthesis, which is a folic acid precursor, or to the acquisition of resistance genes coding for resistant forms of the enzyme (Franklin and Snow, 2005). Resistance of weissellas to sulfonamides was reported by Byrne-Bailey et al. (2009) in *Weissella* spp. isolated from un-amended pig slurry and relied on the presence of *sul1* and *sul2* resistance genes. Figueiredo et al. (2012) isolated sulfonamide-resistant *W. ceti* that caused outbreaks characterized by acute haemorrhagic septicaemia and high mortality rates in rainbow trout. However, no genetic elements responsible for this resistance were described. *Weissella* genomes investigated in this study showed the presence of sulfonamide-resistance genes in *W. ceti* strains WS08 (WS08_0966), WS74 (WS74_1032), and WS105 (WS105_1028) isolated from rainbow trout (Figueiredo et al., 2012). Overall, resistance to vancomycin, fosfomycin, and sulfonamides appears to be a common trait in the genera *Weissella* and *Leuconostoc*. On the other hand, it is frequent that bacterial resistance to sulfonamides often co-exists with trimethoprim resistance, since this substance is used in combination with sulfonamides to minimize bacterial resistance. The known *dfr* trimethoprim resistance genes, which are usually associated with integrons (Skold, 2001) could, however, not be detected in any of the *Weissella* genome sequences analyzed here. Thus, the reported resistance of *W. confusa* (Fairfax et al., 2014; Medford et al., 2014) to trimethoprim may be caused by other modifications than those produced in the target enzyme dihydrofolate reductase (Dfr) that is encoded by the *dfr*-genes.

Weissellas are generally susceptible toward tetracycline. Screening of the published genomes revealed the presence of a gene encoding tetracycline (class C) resistance in *W. ceti* WS105 (WS105_0392) and in *W. thailandensis* fsh4-2 (WT2_00189; Table 3).

It is frequent to find resistance to the most widely used chemotherapeutic agent daunorubicin in probiotic bacteria such as lactobacilli (*Lb. acidophilus* NCFM, *Lb. casei* BD11) and bifidobacteria (*Bifidobacterium animalis* subsp. *lactis* Bb-12, *Bifidobacterium longum* subsp. *infantis* ATCC 15697). When the annotated genome sequences of weissellas were screened for daunorubicin resistance genes, ABC transporter-based resistance genes could be found in several *Weissella* spp. [*W. halotolerans* DSM 20190 (G414_RS0101040), *W. hellenica* Wikim14 (TY24_RS06500), and *W. oryzae* SG25 (WOSG25_RS07165)] (Table 3).

Antibiotic resistance is often based on unspecific mechanisms such as efflux pumps, which are widespread throughout evolution in different bacteria and can pump a variety of drugs out the cells (Piddock, 2006). When we screened the annotated genome sequences of *Weissella* spp., several efflux pumps were detected, such as multiple antibiotic resistance protein MarR, multidrug resistance SMR family protein, MFS multidrug transporter, ABC transporter, and a DedA family protein, which was recently shown to be associated with antibiotic resistance (Kumar and Doerrler, 2014).

### Species-Specific Comparison of Biotechnological and Biosafety Issues

Based on our genomic *in silico* investigation, the potential deleterious and beneficial effects for each of the *Weissella* species in view of their application as starter cultures or probiotics are discussed below, highlighting the controversial issue of strains of the same species being industrially important in some cases, while being problematic from a safety point of view in others.

#### Weissella ceti

*Weissella ceti* (Vela et al., 2011) was isolated from different organs of beaked whales (*Mesoplodon bidens*) such as the spleen, kidney, muscle, brain, and lymph. Several strains of *W. ceti* were reported as pathogens in fish, such as *W. ceti* WS08, *W. ceti* WS74, and *W. ceti* WS105 from farmed rainbow trout in Brazil (Figueiredo et al., 2014; Costa et al., 2015), and *W. ceti* NC36 from farmed rainbow trout in the United States (Ladner et al., 2013). This suggests that weissellosis is a rapidly emerging disease in farmed rainbow trout in different geographical locations. Recently, Welch and Good (2013) reported that *Weissella* spp. closely related with *W. ceti* strains on the basis of their 16S rRNA gene sequences (99% identity) from Chinese and Brazilian out breaks were involved in mortality of farmed rainbow trout in the USA. On the other hand, no reports were found on the application of *W. ceti* strains as starter cultures or as probiotics. In this study it was found that *W. ceti* genomes harbored several virulence factors and antibiotic resistance genes, which point toward a pathogenic potential. The industrial application of such strains thus seems problematic and should be considered carefully on a strain to strain basis in the background of a detailed safety evaluation for the presence of antibiotic resistances and virulence determinants.

#### Weissella cibaria

Originally isolated from Thai fermented foods (Björkroth et al., 2002), *W. cibaria* was also isolated from other sources such as sourdough, fermented milk, cheese, fermented vegetables, fermented fish and meat, and also silage (Srionnual et al., 2007; reviewed by Fusco et al., 2015). However, clinical samples have also been a source of *W. cibaria* (Björkroth et al., 2002), with these bacteria being found in human saliva and the vagina, in human and animal feces and milk, and also in human blood and urine (reviewed by Fusco et al., 2015). *Weissella cibaria* has been targeted for use as starter culture in foods for different purposes such as for probiotic effects. In this sense, the dextran produced by dextransucrase from *W. cibaria* JAG8 had potential prebiotic effect for health benefits, stimulating the growth of probiotic bacteria (Tingirikari et al., 2014). Other beneficial effect of *W. cibaria* were suggested to be its capacity to produce higher levels of exopolysaccharides (EPS), which indicates more acid resistance, thus improving its probiotic capacity during passage throughout gastrointestinal tract (Park et al., 2013). Potentially probiotic *W. cibaria* strains were isolated from kimchi and also from goat milk (Elavarasi et al., 2014), and some strains were suggested to have anticancer activity, immune modulating activity, anti-inflammatory activity, antioxidant activity (patent related to the *W. cibaria*’s anticancer activity registered by Cha et al., 2008; Kang et al., 2011; Kwak et al., 2014), antiviral activity (the avian influenza virus; Rho et al., 2009) and anti-obesity effects being suggested to be more effective than the well-known probiotic bacterium *Lb. rhamnosus* GG (LGG; Ahn et al., 2013). It was also reported that the water-soluble polymers produced by *W. cibaria* inhibited biofilm formation by *Streptococcus mutans*, and thus production of such compounds could reduce oral plaque formation by approximately 20.7% *in vivo* and *in vitro* (Kang et al., 2006). Furthermore, the same authors reported that the hydrogen peroxide produced by *W. cibaria* inhibited the growth of the bacterial agent of periodontal disease, *Fusobacterium nucleatum*, and was effective in reducing the production of hydrogen sulfide and methanethiol responsible for the associated foul smell (Kang et al., 2006).

Several reports proposed different strains of *W. cibaria* as starter cultures in different fermentations, and these were based on its antimicrobial capacity. *W. cibaria* strains produce a variety of antagonistic substances, including organic acids and bacteriocins (i.e., weissellicin 110 produced by *W. cibaria* 110; Srionnual et al., 2007), as mentioned above. Wolter et al. (2014) reported that exopolysaccharide-producing *W. cibaria* MG1 might be a suitable starter culture for sourdough fermentation of buckwheat, quinoa and teff flour. *W. cibaria* was successfully tested in a defined semi-liquid sourdough starter (Gaggiano et al., 2007; Ricciardi et al., 2009), and due to its ability to grow at 45°C and produce EPS, which could be used as an alternative to additives for conditioning the textural properties of bread (Di Cagno et al., 2006), it was considered to be a suitable strain for this application. *Weissella cibaria* could also play an important role in meat fermentation. Thongsanit et al. (2009) proposed *W. cibaria* MSS2 as starter culture for the production of *nham* fermented sausage and for kimchi, due to the capacity of these bacteria to produce glutaminase, which is indispensable in food processing. Furthermore, the use of *W. cibaria* as starter for food fermentations promoted the formation of ornithine from arginine, which in turn may have beneficial health effects, such as anti-obesity effect due to high levels of ornithine in fermented foods (Yu et al., 2009).

On the other hand, *W. cibaria* was reported as an emerging pathogen being associated with bacteremia (Kulwichit et al., 2008) and dog ear otitis (Björkroth et al., 2002), and also as food spoilage organism in sliced vacuum-packed cooked ham (Han et al., 2010). In the present study, we showed that *W. cibaria* KACC 11862 harbored in its genome some virulence determinants (hemolysins) and antibiotic resistance genes (fosfomycin, methicillin, and glycopeptide; Tables 2–3). This argues for a detailed investigation on the virulence potential for this species on a strain basis.

#### Weissella confusa

Due to its previous confusion with *viridans* streptococci, most of infections caused by *W. confusa* were underestimated due to the misidentification of this bacterium by commercial identification systems. When the genus *Weissella* was described by Collins et al. (1993), it was shown that *W. confusa* played an important role in human and animal sepsis and also bacteremia (Green et al., 1990; Olano et al., 2001; Björkroth et al., 2002; Flaherty et al., 2007; Shin et al., 2007; Salimnia et al., 2010, 2011; Harlan et al., 2011; Kumar et al., 2011; Lee et al., 2011; Fairfax and Salimnia, 2012), thumb abscess (Bantar et al., 1991), endocarditis (Shin et al., 2007), osteomyelitis (Kulwichit et al., 2007), and recently it was also shown to be involved in infection of a prosthetic joint (Medford et al., 2014). Here, *in silico* analysis of the *W. confusa* LBAE C39-2 genome showed the presence of virulence determinant genes (encoding collagen adhesion, hemolysin, and mucus-binding proteins) and antibiotic resistance genes (fosfomycin, methicillin, and glycopeptide; Tables 2–3).

On the other hand, due to the widespread use of *W. confusa* in fermented foods (sourdough, cereals, vegetables, fermented milk, cheese; reviewed by Fusco et al., 2015), it was proposed as a starter culture, and also as probiotic which provides various beneficial health effects such as the inhibition of *Helicobacter pylori*, a bacterium that causes chronic inflammation and ulcers in the stomach (Nam et al., 2002). Furthermore, *W. confusa* strains isolated from human feces were proposed as potential probiotics (Lee et al., 2012). Other *W. confusa* strains (UI006 and UI007) isolated from traditional dairy foods from Nigeria were proposed as adjunct cultures for the dairy manufacture industry, because of their antagonistic activities against entero- and uro-pathogens (organic acids, ethanol, and hydrogen peroxide), and their lack of toxic compounds (Ayeni et al., 2009, 2011). In this regard Yang (2013) proposed *W. confusa* LK4 isolated from leek kimchi as a functional starter culture for fermentation of leeks. Due to the aforementioned role in human infections, the biotechnological use of *W. confusa* should also be carefully assessed on a strain to strain basis.

#### Weissella koreensis

These bacteria isolated from a Korean fermented food “kimchi” (Lee et al., 2002) was used as starter culture in kimchi fermentations and production of functional foods, since *W. koreensis* OK1-6 has anti-obesity effects in high-fat diet (HF) induced obese mice (Park et al., 2012). Moon et al. (2012) reported the same anti-obesity effect for *W. koreensis* OK1-6, which produced ornithine from arginine, implying its functional role in reducing obesity. Also, Pi et al. (2014) showed that *W. koreensis* 521 was able to prevent and suppress obesity via the inhibition of pre-adipocyte mitogenesis and differentiation. On the other hand, the application of *W. koreensis* as starter culture together with *Ln. citreum* and baker’s yeast in sourdough to make whole wheat bread was also considered as successful, since a good texture and an extended shelf-life of bread could be obtained (Choi et al., 2012). The use of *W. koreensis* as starter culture in broken rice was also found to provide good organoleptic properties to *jeungpyeon*, which is a Korean fermented rice cake (Choi et al., 2013). *Weissella koreensis* was also proposed for use as probiotic in pigs, since dietary supplementation with *W. koreensis* WKG2 in growing pigs could improve the average daily gain (ADG) and could have a beneficial effect on the immune response during an inflammatory challenge (Wang et al., 2014). On the other hand, no negative effects on heath were yet attributed to this species. However, in our *in silico* genome investigation, we showed the presence of potential virulence determinants and antibiotic resistance genes in some strains of *W. koreensis* (Tables 2–3), again indicating that a safety assessment for strains targeted for biotechnological use should be done.

#### Other *Weissella* spp.

Regarding their health-promoting activities, several species of *Weissella* exhibited beneficial effects. *W. kimchii* is a hydrogen peroxide-producing species that has been proposed as probiotic to prevent vaginal infections against *Candida albicans*, *Escherichia coli*, *S. aureus*, and *Streptococcus agalactiae* (Lee, 2005; Lee et al., 2011). *W. hellenica* was isolated from different sources such as fermented vegetables, fermented sausage, fermented fish, cheeses, cow’s milk and flounder intestine (Leong et al., 2013; reviewed by Fusco et al., 2015), and is known for its potential probiotic activity due to its bacteriocin-producing capacity (weissellicins D, L, M, and Y). Strains from this species are active against several pathogens (Masuda et al., 2011; Leong et al., 2013; Chen et al., 2014) and have potential also as bio-protective culture to improve safety and shelf-life of foods like tofu (Chen et al., 2014). *W. hellenica* strains isolated from different sources (cheese, fermented sausage, fermented vegetables, cow’s milk, flounder intestine; reviewed by Fusco et al., 2015) were also reported as glucan (EPS) producers (Kim et al., 2008), which may have industrial applications. Finally, *W. paramesenteroides* isolated from soil, vegetables, cheese, fermented sausage, fermented sea food, fermented vegetables and also from feces, cow’s milk, and gut of rainbow (reviewed by Fusco et al., 2015) can have antibacterial activity by production of bacteriocins such as weissellin A from *W. paramesenteroides* DX (Papagianni and Papamichael, 2011), a bacteriocin from *W. paramesenteroides* DFR-8 (Pal and Ramana, 2010), and also non-proteinaceous antibacterial compounds (Pal and Ramana, 2009) with a broad inhibitory spectrum against Gram-positive and Gram-negative bacteria. These strains may therefore be interesting for their use as food biopreservatives. On the detrimental side, Han et al. (2010) showed that *W. paramesenteroides* was involved in sliced vacuum-packed cooked ham spoilage.

Due to their heterofermentative metabolism, some weissellas are also involved in food spoilage and lead to sensory defects of, e.g., meat products (Marsden et al., 2009). In this regards, *W. viridescens* plays an important role in meat spoilage, producing a greenish slime on meat surfaces as a result of its production of H_2_O_2_. In cooked hams, formation of cavities can result from the production of CO_2_ by these bacteria. Furthermore, taking into account data presented here from *in silico* analyses of *Weissella* spp. genomes, the presence of virulence determinants, especially hemolysins, were detected in all strains analyzed. Also antibiotic resistance genes, such as those coding for fosfomycin and methicillin resistances (Tables 2–3), occur in this species. Also in this case, therefore, the safety of strains intended for industrial use should be investigated for each strain in detail.

## Conclusion

Considering the large number of health-promoting benefits which could arise from the use of strains of *Weissella* spp., such as antibacterial, anti-viral, anti-tumoral, anti-obesity, anti-inflammatory, and antioxidant activities, several weissellas could be targeted for use as starter cultures or probiotics. By contrast, specific strains of *Weissella* species have also been involved as pathogens in the etiology of different diseases such as bacteremia, endocarditis, sepsis, and may even cause mortality. In fact, the safety of this genus has not been deeply studied, as only some strains are considered as opportunistic pathogens. Thus, the application of strains in foods and feeds or for humans as probiotics should be done with caution. In this report we showed that screening of genome sequences revealed the presence of several virulence and antibiotic resistance genes, which could be the basis of the potential pathogenicity of some strains. However, the presence of single determinants should not be an exclusion criterium for weissellas that may have overall beneficial effects. Thus, selection of weissellas intended to be used as starters or as probiotics should be investigated carefully regarding their safety aspects, preferably by genome sequencing an annotation, since the heterogeneity in nucleotide sequences of well-known virulence and antibiotic resistance-genes make these undetectable by PCR methods. Generally, the application of *W. confusa* and *W. cibaria* strains as starter cultures or as probiotics should be approached with caution, carefully selecting strains which lack pathogenic potential and which do not possess transferable antibiotic resistance genes.

### Conflict of Interest Statement

The authors declare that the research was conducted in the absence of any commercial or financial relationships that could be construed as a potential conflict of interest.

## Footnotes

1 http://www.bacterio.net

2 http://www.ncbi.nlm.nih.gov/gene
